# Microstructural Origin of the Double Yield Points of the Metallocene Linear Low-Density Polyethylene (mLLDPE) Precursor Film under Uniaxial Tensile Deformation

**DOI:** 10.3390/polym13010126

**Published:** 2020-12-30

**Authors:** Obaid Iqbal, Jean Claude Habumugisha, Shengyao Feng, Yuanfei Lin, Wei Chen, Wancheng Yu, Liangbin Li

**Affiliations:** 1 National Synchrotron Radiation Laboratory, Anhui Provincial Engineering Laboratory of Advanced Functional Polymer Film, CAS Key Laboratory of Soft Matter Chemistry, University of Science and Technology of China, Hefei 230026, China; obaid@mail.ustc.edu.cn (O.I.); hab@mail.ustc.edu.cn (J.C.H.); fsyfys@mail.ustc.edu.cn (S.F.); linyuanf@mail.ustc.edu.cn (Y.L.); wc003@ustc.edu.cn (W.C.); 2 South China Advanced Institute for Soft Matter Science and Technology, School of Molecular Science and Engineering, Guangdong Provincial Key Laboratory of Functional and Intelligent Hybrid Materials and Devices, South China University of Technology, Guangzhou 510640, China

**Keywords:** mLLDPE, double yield points, in situ SAXS/WAXS

## Abstract

The microstructural origin of the double yield points of metallocene linear low-density polyethylene (mLLDPE) precursor films has been studied with the assistance of the synchrotron radiation small- and wide-angle X-ray scattering (SAXS/WAXS). It has been shown that the microstructural origin of the double yield points is highly related to the initial orientation of the original precursor film. For less oriented mLLDPE precursor films, the rearrangement of lamellae and the appearance of the monoclinic phase are the microstructural origins of the first yield point. In comparison, for the highly-oriented mLLDPE precursor film, only the orthorhombic-monoclinic phase transition appears at the first yield point. The melting-recrystallization and the formation of the fibrillary structure happen beyond the second yield point for all studied mLLDPE precursor films. Finally, the detailed microstructural evolution roadmaps of mLLDPE precursor films under uniaxial tensile deformation have been established, which might serve as a guide for processing high-performance polymer films by post-stretching.

## 1. Introduction

The vast majority of polymer films or fibers undergo external stretching at the initial stage of processing, such as the extrusion-casting and spinning [1,2]. Pre-stretched polymer films or fibers usually exhibit superior properties, such as the substantially enhanced tensile strength along the oriented direction. One typical case is the extremely high modulus of polymer fiber due to the highly-oriented chains along the spinning direction [3,4,5]. Furthermore, post-stretching processing is usually indispensable for polymer films or fibers with specific applications [6,7]. For instance, the polyethylene (PE) and polypropylene (PP) microporous films used as battery separators are obtained by the post-stretching of polymer precursor films to create micro-pores [8,9]. Since the modulus of the amorphous domain is smaller than that of the crystalline domain, micro-pores are generally located in the amorphous domain of PE. Therefore, the ultimate properties of polymer films are closely related to the microstructural evolution of both crystalline and amorphous domains during stretching [10,11,12,13,14].

The uniaxial deformation of glassy or semicrystalline polymers usually leads to a single yield point. In a pioneering work studying the mechanical properties of ethylene copolymers and branched polyethylene under tensile deformation at room temperature, Popli and Mandelkern presented experimental evidence of the existence of the double yield points for the first time and attributed it to the broad distribution of lamella thickness [15]. Since then, it aroused a great research interest in the community of polymer physics to elucidate the microstructural mechanisms of the double-yielding phenomenon [16,17,18,19,20,21,22,23,24,25,26,27,28,29,30,31]. Séguéla et al. proposed that the crystal slip in the mosaic crystalline structure and the shear of the crystalline domains are responsible for the two yield points, respectively [16,17]. Brooks et al. conducted a series of experiments to figure out the origin of the double yield points [18,19,20]. The first yield point was proposed to be associated with the recoverable reorientation or rearrangement of lamellae, while the second one was attributed to the fragmentation of lamellae by *c* shear [18]. Lucas et al. investigated the origin of the second yield point from the view of melting and recrystallization [21]. It should be also pointed out here that the double-yielding phenomenon has been observed in many other polymeric systems, such as polyamide 6 [32,33], poly(ethylene terephthalate)/polyethylene and polycarbonate/polyethylene blends [34,35,36], ethylene/methacrylic acid copolymers [37], polypropylene [38,39], to name just a few.

Undoubtedly, these previous works have provided us with a profound understanding of the microstructural origin of the double yield phenomenon. However, there are still several problems that remain on the double yield phenomenon which has to be answered. For example, how does the different pre-orientation of the semi-crystalline polymers affect the origins of the double yield points due to their complex initial structure distributions? To clarify this problem, in this work, the mechanical properties of metallocene LLDPE (mLLDPE) precursor films with different orientations under uniaxial tensile deformation were studied. By in situ synchrotron radiation X-ray scattering, real-time monitoring of the microstructural evolution during the deformation was accessible. Specifically, a combination of wide- and small-angle X-ray scattering (WAXS/SAXS) allowed us to detect the variations in the crystal structure at the nanoscale, as well as the lamellae at the sub-micron length scale. Based on 2D WAXS, it is found that the polymorphic transition or the appearance of the monoclinic phase happens for all mLLDPE precursor films with different orientations at the first yield point. The four-spot pattern appears in 2D SAXS patterns for less oriented mLLDPE precursor films, whereas no significant change is observed for the highly-oriented one. The strain hardening point is characterized by the inflection point in the stress–strain curves, where the lamellae slip occurs as indicated by the decreasing lateral size of (200) and (001) crystallographic planes from WAXS. As the strain increases to the second yield point, the melting-recrystallization phenomenon is observed as evidenced by the abrupt change of the long period around diagonal direction referring to the stretching direction. Moreover, for all precursor films, the fibrillar structure is formed as suggested by the disappearance of diagonal peaks in 2D SAXS patterns and the increment of the long period along the equatorial direction. The hierarchically structural transition influenced by the orientation of initial precursor films under uniaxial tensile deformation clearly shows the critical role of the crystalline domain in determining the nonlinear mechanical property.

## 2. Experiments

### 2.1. Materials and Sample Preparation

mLLDPE was supplied by ExxonMobil, with an average butyl branch content of 1.2 mol%. The polydispersity index $M_{w}$/$M_{n}$ and the number average molecular weight $M_{n}$ are 3.9 and 24 kg/mol, respectively. A homemade single screw extrusion-casting machine was used to prepare mLLDPE films with different draw ratios with a die width of 160 mm and a thickness of 1 mm, respectively. The temperatures of extrusion were fixed at 170, 180, 190, and 200 ${}^{\circ }$C, and the die temperature was set at 180 ${}^{\circ }$C. In addition, an air knife was mounted close to the exit of the die to enhance the cooling of the extruded mLLDPE melt. The temperature of the casting and cooling roll was set as 80 and 70 ${}^{\circ }$C, respectively.

### 2.2. Characterization

#### 2.2.1. In Situ SAXS/WAXS

The in situ two-dimensional (2D) SAXS and WAXS experiments were carried out at the beamline BL19U2 in Shanghai Synchrotron Radiation Facility (SSRF) in the combination with a custom-built uniaxial stretching machine. The X-ray wavelength at BL19U2 is 0.103 nm. A Pilatus 1M detector (1043×981 pixels with a pixel size of 172 μm) and a Pilatus 2M detector (1475×1679 pixels with a pixel size of 172 μm) were used to collect the time-resolved SAXS and WAXS scattering patterns. The acquisition time for SAXS and WAXS is 1 s and 5 s, respectively. The original length of the sample between clamps was 25 mm, and the stretching rate during deformation was fixed at 0.1 mm/s. All experiments were conducted at ambient temperature (25 ${}^{\circ }$C). The sample-to-detector distances were calibrated to be 3050 mm and 196 mm for SAXS and WAXS, respectively. 2D WAXS and SAXS scattering patterns were analyzed by Fit2D software developed by the European Synchrotron Radiation Facility (ESRF) [40].

The 1D SAXS scattering profiles were obtained by integrating the 2D SAXS patterns as a function of the module of the scattering vector q=4πsinθ/λ. The long period of the sandwiched lamellar structure is defined as the sum of the average thickness of amorphous and crystal layers: L=2π/q, which can be calculated according to Bragg’s equation with *q* being peak positions of I(q).

The crystallinity χ of mLLDPE precursor films was obtained by the multi-peak deconvolution of 1D integrated WAXS curves. The relative contents of the orthorhombic phase $\chi_{O}$ and the monoclinic phase $\chi_{M}$ were calculated according to Equations (1) and (2), respectively:

(1) $$\chi_{O}=\frac{\sum I_{O}}{\sum I_{O}+\sum I_{M}+\sum I_{amor}}\times 100\%,$$

(2) $$\chi_{M}=\frac{\sum I_{M}}{\sum I_{O}+\sum I_{M}+\sum I_{amor}}\times 100\%.$$

Here, $I_{O}$, $I_{M}$, and $I_{amor}$ is the peak area of the orthorhombic, monoclinic, and amorphous phases (The subscripts *O*, *M* and *amor* refer to the orthorhombic, monoclinic, and amorphous phase, respectively).

The orientation of the crystal lamellae was calculated by Hermans’ orientation function, $O_{LJ}$:

(3) $$O_{LJ}=\frac{3\langle \cos^{2}\varphi \rangle -1}{2},$$

where φ is the angle between the normal direction of the crystallographic plane and the reference direction (tensile direction).

The structural information of initial mLLDPE precursor films with different draw ratios before stretching is summarized in Table 1. Four pre-oriented samples used in this study are named PE-61, PE-72, PE-78, and PE-87 according to the orientation parameter $O_{LJ}$, calculated from the SAXS patterns. The draw ratio, crystallinity χ (%), and the long periods of lamellae stacks in the meridian $L_{m}$ and diagonal $L_{d}$ are also presented.

#### 2.2.2. Scanning Electron Microscope (SEM) Measurements

A field emission scanning electron microscope (Gemini-SEM 500) was employed to characterize the surface morphology of the initial samples before stretching using an accelerating voltage of 2 kV. To remove the amorphous phase, etching of the precursor films for 10 min was performed before observing the surface morphologies. For etching, 25 mL of concentrated sulfuric acid, 0.4 g of potassium permanganate, and 25 mL of concentrated nitric acid solution was prepared by quick stirring and careful mixing. Then, mLLDPE precursor films were dissolved in the solution. After reaction, the films were washed according to the method proposed by Olley and Bassett [41]. To enhance electrical conductibility before testing, the samples were sputter-coated with a gold ion beam for 20 s.

## 3. Results

### 3.1. Crystal Morphologies

**Figure 1:**
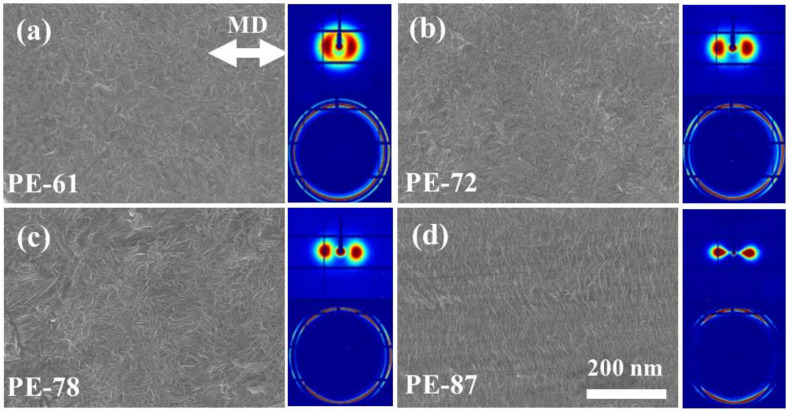
SEM images together with corresponding 2D SAXS and WAXS patterns of mLLDPE precursor films. The scale bar is the same for all micrographs.

Figure 1 presents the SEM images of different mLLDPE precursor films and the corresponding 2D SAXS and WAXS patterns. PE-61 shows an unapparent oriented lamellar structure. This is also reflected by the two broad arcs in the 2D SAXS patterns and the nearly isotropic scattering rings in the 2D WAXS patterns. The differences in the SEM images of PE-61, PE-72, and PE-78 are nearly indistinguishable to the naked eye but can be identified from the narrowed arcs in 2D SAXS. In comparison, highly-oriented lamellae perpendicular to the machine direction (MD) can be seen from the SEM image of PE-87. For the 2D WAXS pattern, the two scattering rings assigned to (110) and (200) crystallographic planes concentrate to the equatorial direction, while no scattering signal is observed along the meridian direction. Overall, with the increasing draw ratio, the chain orientation gets significantly enhanced.

### 3.2. Mechanical Property

**Figure 2:**
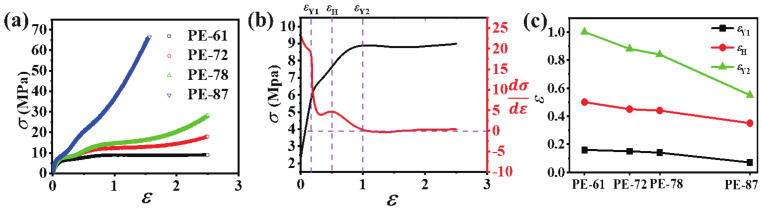
(**a**) Engineering stress–strain curves of mLLDPE precursor films with different orientations; (**b**) the first derivative of the stress–strain curve of PE-61 together with the definition of the double yield points and the strain hardening point; (**c**) the double yield points and the strain hardening point for four different mLLDPE precursor films.

Figure 2a shows the engineering stress–strain (σ-ε) curves of different mLLDPE precursor films. Two yield points can be discerned for all mLLDPE precursor films, even for the highly-oriented one PE-87. To obtain a quantitative analysis, the first derivative of the stress–strain curve, $\sigma^{\prime }\left(\varepsilon \right)=d\sigma /d\varepsilon$ was plotted in Figure 2b. Here, the stress–strain curve of PE-61 is given as a typical example. Two yield points are defined as the starting points of the decrement of $\sigma^{\prime }\left(\varepsilon \right)$. In this way, the first yield point at $\varepsilon_{Y1}=0.19$ and the second yield point at $\varepsilon_{Y2}=1$ are obtained, respectively. The strain hardening point ($\varepsilon_{H}=0.5$) locating between the two yield points is defined as the inflection point, where a local maximum of $\sigma^{\prime }\left(\varepsilon \right)$ is reached. Thus, the σ−ε curve can be divided into four regions. In the linear elastic region I ($0<\varepsilon <\varepsilon_{Y1}$), the stress increases linearly with the strain, and the deformation is reversible. In region II ($\varepsilon_{Y1}<\varepsilon <\varepsilon_{Y2}$), $\sigma^{\prime }\left(\varepsilon \right)$ decreases to a value of 4.8 MPa first and then increases to a local maximum of 5 MPa at $\varepsilon_{H}$. In region III ($\varepsilon_{H}<\varepsilon <\varepsilon_{Y2}$), $\sigma^{\prime }\left(\varepsilon \right)$ decreases almost to zero gradually. In region IV ($\varepsilon_{Y2}<\varepsilon <2.5$), $\sigma^{\prime }\left(\varepsilon \right)$ reaches a plateau. The second yield point becomes unclear for highly-oriented PE-87, where no clear local maximum stress is observed. Figure 2c summarizes the above three transition points for different mLLDPE precursor films. With the increasing initial orientation, all three of the transition points decrease, especially for the second yield point. As mentioned above, the yield point is highly related to the microstructural evolution, especially the crystalline domain. Therefore, in the following section, the structural information under uniaxial tensile deformation, especially at the vicinity of two yield points, is characterized in detail based on in situ SAXS/WAXS results.

### 3.3. 2D SAXS/WAXS Patterns

**Figure 3:**
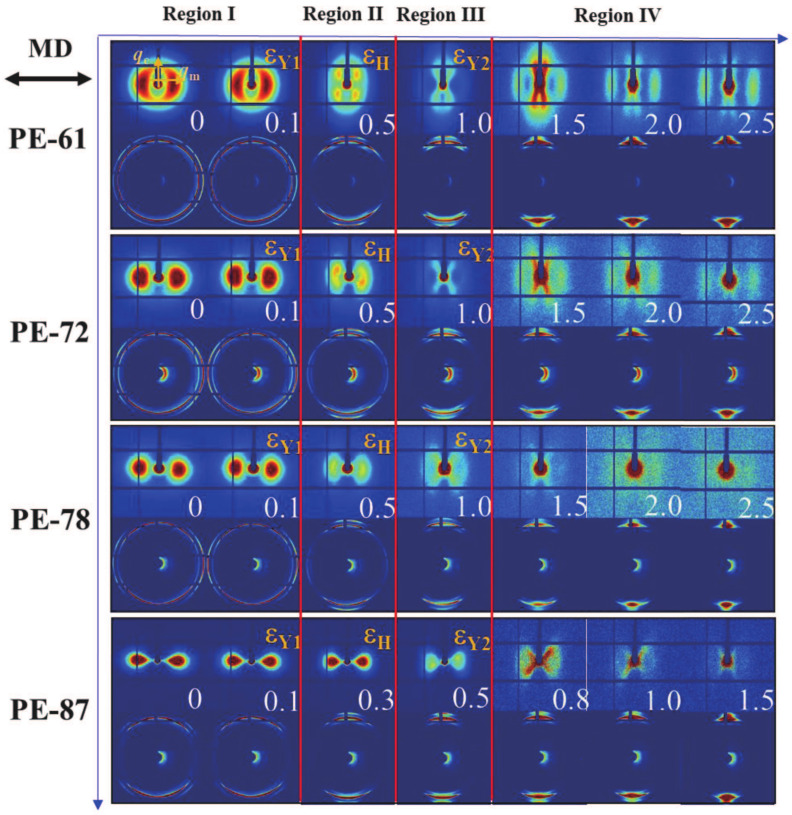
Representative 2D SAXS and WAXS patterns for mLLDPE precursor films with different orientations during the uniaxial tensile deformation. The corresponding strain is shown in the right corner of the 2D SAXS pattern. The tensile direction is horizontal.

Figure 3 shows the 2D SAXS and WAXS patterns of different mLLDPE precursor films at different strains. For the less oriented films, such as PE-61, there are two broad arcs along the meridian direction in the 2D SAXS pattern, while two nearly isotropic scattering rings exist in the corresponding 2D WAXS patterns. This suggests the oriented lamellae is perpendicular to the machine direction (MD). Beyond the first yield point, the four-spot patterns appear in the 2D SAXS, together with the concentrated scattering signal along the equatorial direction in 2D WAXS. The appearance of four-spot patterns in SAXS is indicative of the staggered roof structure or rearrangement of lamellae along the diagonal direction. By contrast, the four-spot pattern does not appear beyond the first yield point for PE-87, which might be attributed to the highly-oriented lamellae in the original film. For all the studied mLLDPE precursor films, as the strain gets beyond the second yield point, a new scattering appears in the 2D SAXS. This indicates the formation of the fibrillar structure [11].

### 3.4. Strain Dependent Long Period and Crystallinity

The microstructural parameters, such as the long period and crystallinity, can be calculated from the 1D SAXS and WAXS profiles directly. This allows us to monitor the microstructural evolution of mLLDPE precursor films during the deformation. Again, the less oriented PE-61 is taken as a typical example to illustrate how these microstructural parameters change as a function of the strain.

**Figure 4:**
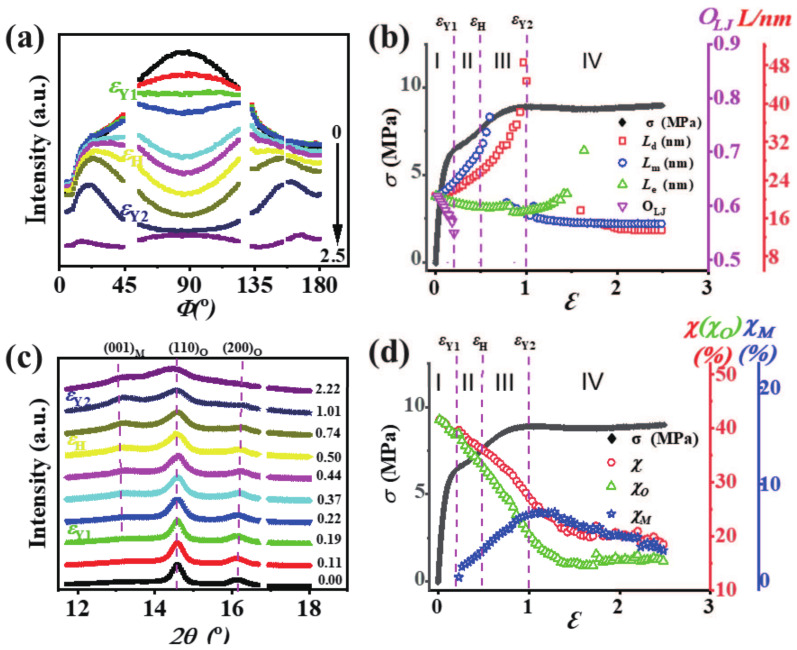
(**a**) Strain dependent SAXS azimuthal integration profiles for PE-61; (**b**) evolution of the long periods along the meridian (blue circles), diagonal (red square), equatorial (green triangle) directions, and the corresponding orientation (pink triangle) as a function of the strain; (**c**) 1D integrated WAXS curves as a function of the strain; (**d**) evolution of the overall crystallinity (red circle), and the relative contents of the monoclinic (blue stars) and the orthorhombic phases (green triangle) as a function of the strain. The engineering stress–strain curve is also plotted for the correlation between the microstructural evolution and mechanical behavior.

Figure 4a shows the strain-dependent azimuthal integrated SAXS curves, where the single peak evolves into two peaks and finally becomes a single one again. Meanwhile, the peak gets slightly broadened with the increasing strain, suggesting that inter or intra-lamellar slips occur around the first yield point. The two peaks beyond the first yield point indicate the reorientation or rearrangement of lamellae under uniaxial deformation. Since the original film is anisotropic, the long periods along different directions can provide more detailed information on the rearrangement of lamellae during the deformation. In Figure S2, the 1D integrated SAXS intensity distribution profiles were taken within a small region along the meridian direction, the diagonal direction, and the equatorial direction. In this way, the corresponding long periods $L_{m}$, $L_{d}$, and $L_{e}$ can be obtained readily. As plotted in Figure 4b, their evolutions with the strain are coupled with the stress–strain curve to establish a direct relationship between the microstructure and mechanical property. The initial value of $L_{m}$, $L_{d}$, and $L_{e}$ is about 20 nm; however, as the deformation develops, they display distinct variation trends. $L_{m}$ increases rapidly from the initial 20.56 nm to a maximum of 37.2 nm just beyond the strain hardening point. The orientation of the lamellae stacks $O_{LJ}$ decreases abruptly from 0.61 to 0.54 around the first yield point that corresponds to the initiation of a four-spot pattern or rearrangement of lamellae stacks. After that, the scattering signal along the meridian direction disappears suddenly in a narrow range of strain but reappears soon. The long period along the meridian direction at this time is much shorter with an average value of 15 nm.

For the long period along the diagonal direction, $L_{d}$ exhibits a similar trend as $L_{m}$. Namely, it increases from the initial 20.76 nm to a maximum of 48.5 nm around the second yield point. A rapid increase of $L_{d}$ in region III ($\varepsilon_{H}<\varepsilon <\varepsilon_{Y2}$) indicates the destruction of the lamellae stacks. After the second yielding, the long period along the diagonal direction vanishes in a stain range from about 1 to 1.5, and then emerges at ε>1.5 with a much smaller value of 14 nm. For the long period along the equatorial direction $L_{e}$, it remains almost invariant before the second yield point, followed by a rapid increase in region IV where the fibrillar structure forms. The vanishing of the two peaks beyond the second yield point, together with the widened peak shown in the azimuthal integrated SAXS curves (see Figure 4a) also strongly suggests that the melting or destruction of the initial lamellae, and the further formation of a new lamellar or fibrillar structure until the uniaxial deformation ceases.

The WAXS results provide crystallographic information, such as the crystal phase and crystallinity. As shown in Figure 4c, two diffraction peaks assigned to (110) and (200) crystallographic plane of the orthorhombic phase can be discerned at the very beginning of the deformation, denoted as ${\left(110\right)}_{O}$ and ${\left(200\right)}_{O}$. With the strain increasing to the first yield point, a new peak assigned to the (001) crystallographic plane of the monoclinic phase (denoted as ${\left(001\right)}_{M}$) appears, and its intensity is enhanced gradually with the increasing strain. With the proceeding of the deformation, the peak broadening for all crystallographic planes is observed around the second yield point $\varepsilon_{Y2}$ = 1. It indicates the lattice distortion as well as the formation of fibrillary structure, which are in good accordance with the SAXS results. The multi-peak deconvolution was employed to extract the crystallinity of different crystal phases as shown in Figure S4. The total crystallinity χ, the fraction of the orthorhombic phase $\chi_{O}$ and the monoclinic phase $\chi_{M}$ for PE-61 are presented in Figure 4d. Before the first yield point, $\chi_{O}$ reduces from 41% to 39% due to either the fragmentation of the crystals or the inter lamellae crystal slip. Beyond the first yield point, the strain-induced polymorphic transition is observed. The emerging of the crystallographic planes ${\left(001\right)}_{M}$ and ${\left(201\right)}_{M}$ in the 2D WAXS patterns verify the formation of the monoclinic phase. In region II ($\varepsilon_{Y1}<\varepsilon <\varepsilon_{H}$), χ and $\chi_{O}$ decrease substantially while $\chi_{M}$ increases. Note that, in this region, the increment in $\chi_{M}$ is approximately equal to the decrement in $\chi_{O}$. In region III ($\varepsilon_{H}<\varepsilon <\varepsilon_{Y2}$), the variation trend of χ, $\chi_{O}$, and $\chi_{M}$ remains nearly unchanged; However, the decrement in $\chi_{O}$ is larger than the increment in $\chi_{M}$. After the second yield point, $\chi_{M}$ starts to decrease as a result of the stretch-induced melting of crystals. The crystallinity $\chi_{O}$ reaches a plateau with an average value of 15% after the strain ε≈1.5, possibly due to the formation of the fibrillar crystals [42].

### 3.5. Influence of Different Orientations of mLLDPE Precursor Film

**Figure 5:**
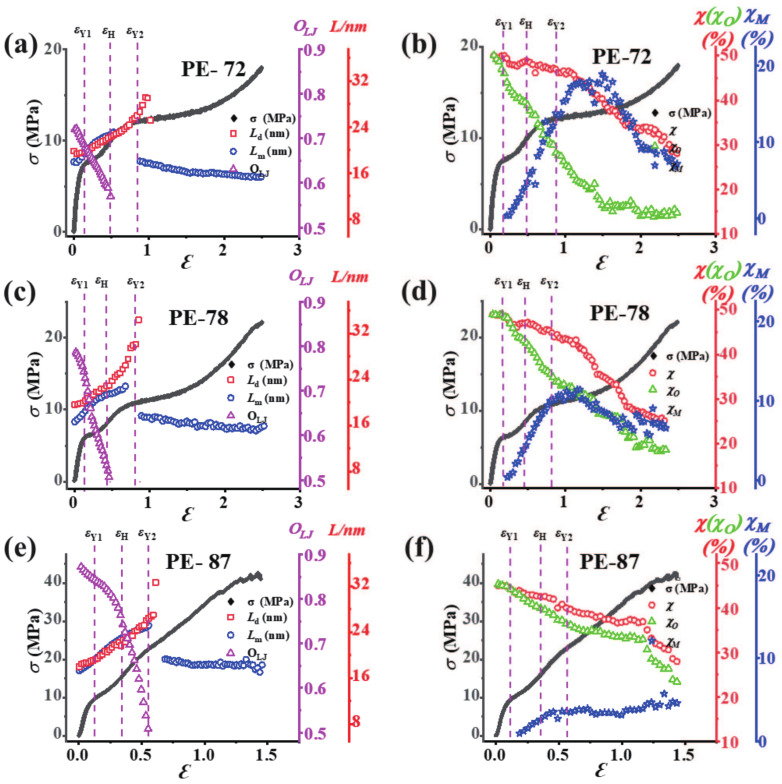
The long periods along the meridian and diagonal direction and the Hermans’ orientation factor for (**a**) PE-72, (**c**) PE-78, and (**e**) PE-87 films. The total crystallinity and the relative contents of the monoclinic and orthorhombic phases for (**b**) PE-72, (**d**) PE-78, and (**f**) PE-87 films.

To clarify the effects of the initial orientation of mLLDPE precursor film on the microstructural evolution, we present the evolution of the long periods and the crystallinity of different mLLDPE precursor films under uniaxial tensile deformation in Figure 5. The PE-72 and PE-78 show quite similar trends. $L_{m}$ for both samples (having an initial value of 17.94 nm and 16.6 nm for PE-72 and PE-78) increases linearly with the strain and reaches a maximum before the second yield point (with the maximum of 23.15 nm and 22.8 nm for PE-72 and PE-78). Then, around the second yield point, a new $L_{m}$ with 15 nm is obtained for both PE-72 and PE-78. For $L_{d}$, no significant variations are observed in region I. In regions II and III, $L_{d}$ increases rapidly to a maximum (29.1 nm for PE-72, 34.37 nm for PE-78). Beyond the second yield point, the long period along with the diagonal direction vanishes, which might be attributed to the increasing lamellar orientation in the mLLDPE precursor films.

The total crystallinity together with the relative contents of the orthorhombic and monoclinic phases are summarized in Figure 5b,d, and 5f for PE-72, PE-78, and PE-87 films. The total crystallinity χ for both PE-72 and PE-78 films decreases slightly (almost 1% due to crystal destruction) before the first yield point, and it follows a continuous decline until the second yield point (about 4 to 5% due to the crystal deformation as a result of shearing or slipping). Beyond the second yield point, an abrupt reduction in χ about 18–20% is observed, suggesting the severe destruction of the crystal lamellae and the simultaneous transformation into the fibrillary structure. Interestingly, the main difference between PE-72 and PE-78 is the relative content of the monoclinic phase. PE-72 has a highest monoclinic fraction about 17% around the second yield point, while for PE-78 the monoclinic fraction is only about 10%.

As shown in Figure 1d, the lamellae are highly-oriented perpendicular to the machine direction in the PE-87 film. The film exhibits a hard elastic property due to the tightly-packed lamellae, which is confirmed by the step-cyclic deformation experiments (given in Figure S5). The long periods ($L_{m}$ and $L_{d}$) and orientation factor ($O_{LJ}$) calculated from the SAXS results as well as the engineering stress–strain are plotted in Figure 5e. During the deformation, $L_{m}$ and $L_{d}$ show almost the same trend, i.e., an increase from 17.32 nm to a maximum of 25.1 nm for $L_{m}$ and from 18 nm to a maximum of 27 nm for $L_{d}$ around the second yield point. In a small range of the strain after the second yield point, the scattering peak along the meridian direction disappears, indicating the melting of original lamellae. Afterwards, the reappearance of this scattering peak with a corresponding value of $L_{m}\approx$ 18 nm suggests a recrystallization phenomenon. $O_{LJ}$ for PE-87 film shows a different trend as compared with that of other less oriented precursor films. It decreases gradually from 0.87 to 0.76 till the strain hardening point ($\varepsilon_{H}=0.35$), followed by a sudden decrease from 0.76 to 0.50 till the second yield point. The final new lamellae suggest the formation of the fibrous crystals as the long period values coincide with the initial one. Figure 5f shows that both χ and $\chi_{O}$ start to decline after the first yield point, where χ decreases from 44% to 40% around the second yield point and later decreases to 27% till the end of the deformation. The relative content of the monoclinic phase $\chi_{M}$ is quite small after the first yield point and approaches a plateau with an average value of 3.6% gradually till the deformation finishes. Last but not least, for the highly-oriented precursor film PE-87, the stretch-induced polymorphic transition (refer to the orthorhombic-monoclinic phase transition in this work) observed in less oriented ones becomes inconspicuous around the first yield point; moreover, the fiber slipping in the tensile direction around the second yield point dominates the melting and recrystallization [42,43].

### 3.6. Micro-Strain and Lateral Size Evolution

**Figure 6:**
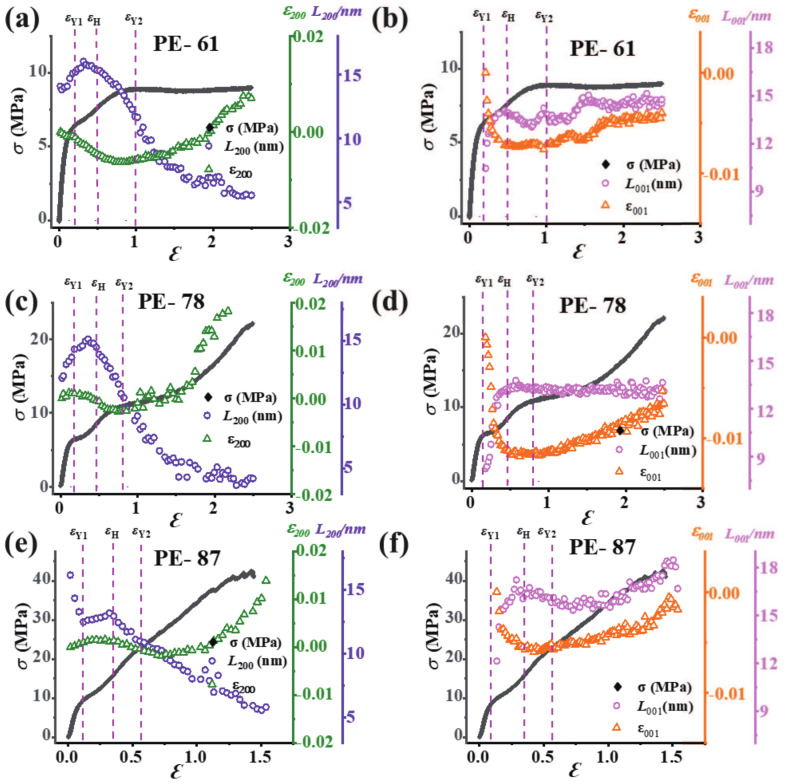
The lateral size and the micro-strain of the ${\left(200\right)}_{O}$ crystallographic plane (**a**,**c**,**e**) and the ${\left(001\right)}_{M}$ crystallographic plane (**b**,**d**,**f**) for different mLLDPE precursor films. The engineering stress–strain curves are also drawn to link the mechanical behavior with microstructure.

The crystalline domain starts to bear the external force at the large strain region. To check the micro-deformation of the lamellae, the lateral size *L* and the corresponding micro-strain ε were calculated as summarized in Figure 6. By applying the Gaussian peak fitting on 1D integration profiles in the equatorial region (using mask protocol in a range from 1${}^{o}$ and 5${}^{o}$), the evolution of the crystal lateral sizes ($L_{200}$ and $L_{001}$) and micro-strains ($\varepsilon_{200}$ and $\varepsilon_{001}$) (by using the crystal *d*-spacing of $d_{200}$ and $d_{001}$) for the crystallographic planes ${\left(200\right)}_{O}$ and ${\left(001\right)}_{M}$ were obtained. *d* and *L* were calculated according to Bragg’s equation and Scherrer’s equation [7], respectively.

As the ${\left(200\right)}_{O}$ plane is perpendicular to the main stress in the equatorial region, it displays elastic straining parallel to the main stress. Because of the low elastic constant, their spacing and block size of the ${\left(200\right)}_{O}$ plane are expected to be sensitive to the local stress [44]. Considering that the general trend is quite similar for less oriented films (PE-61, PE-72, and PE-78), only PE-61 is discussed here as a representative. $\varepsilon_{200}$ increases in region I and reaches a maximum value of 16 nm in region II. Afterward, a continuous decrease in $L_{200}$ is observed with an average size of 5 nm at the final strain of 2.5. The micro-strain $\varepsilon_{200}$ decreases continuously in the regions I and II and reaches a minimum value of −0.0063 in region III. The polymorphic transition after the first yield point leads to the releasing of the stress. This explains the decrement in both $L_{200}$ and $\varepsilon_{200}$ in region II. Around the second yield point $\varepsilon_{Y2}$, the increment in $\varepsilon_{200}$ and the decrement in $L_{200}$ indicate explicitly the crystal destruction. For the highly-oriented PE-87, $L_{200}$ decreases rapidly from 16.1 nm to 12.4 nm in region I, which should be attributed to the imperfect alignment of lamellae along the tensile direction. In region II, $L_{200}$ increases slightly to 13.1 nm followed by a continuous decline in regions III and IV, whose trend is similar to the above less oriented films. Besides the orthorhombic phase, the deformation of the monoclinic phase is also crucial to understand the origin of the yield. Since the monoclinic phase appears beyond the first yield point, the quantitative analyses are performed in regions II–IV. As shown in Figure 6b,d,f, the general trend for both $L_{001}$ and $\varepsilon_{001}$ is quite similar for less and highly-oriented mLLDPE precursor films. A significant increase in $L_{001}$ and a decrease in $\varepsilon_{001}$ are observed in region II. Then, they remain almost constant in region III. In region IV, slight increases are observed, which is in line with the macroscopic stress–strain curve.

## 4. Discussion

Since all experiments were conducted at room temperature well above the glass transition temperature, the origin of the double yield points should be related to the crystalline phase or crystallite network. As mentioned above, the stress–strain curve can be divided into four regions: (a) linear elastic region I ($0<\varepsilon <\varepsilon_{Y1}$); (b) region II ($\varepsilon_{Y1}<\varepsilon <\varepsilon_{H}$) includes the first yielding and the stress plateau, (c) region III ($\varepsilon_{H}<\varepsilon <\varepsilon_{Y2}$) includes the strain hardening and the second yielding, (d) the final strain hardening region IV ($\varepsilon >\varepsilon_{Y2}$). Based on the microstructural parameters ($L_{m}$, $L_{d}$, $O_{LJ}$, χ, $\chi_{O}$, $\chi_{M}$, $L_{200}$, $L_{001}$, $\varepsilon_{200}$ and $\varepsilon_{001}$) and the initial lamellae orientation, the microstructural origin of the double yield points can be clarified. Here, several interesting findings are summarized: (i) For less oriented precursor films like PE-61, the first yield point is associated with the rotation of the lamellae stacks and polymorphic transition, while the second one is related to the lamellar fragmentation and melting-recrystallization; (ii) for the highly-oriented precursor film PE-87, the first yield point is characterized by the polymorphic transition without the rotation of lamellae, while the second one is closely related to the lamellar fragmentation and melting-recrystallization, which is the same as less oriented precursor films.

**Figure 7:**
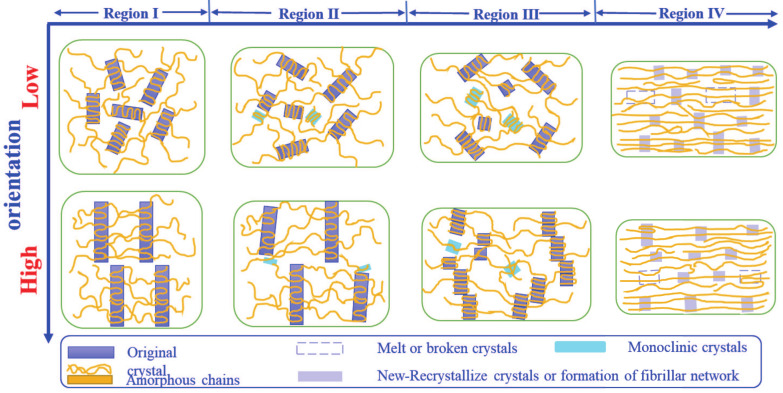
Schematic illustration of the microstructural deformation mechanisms for less and highly-oriented precursor films. The tensile direction is horizontal.

The microstructural evolution pathways of lamellar stacks for mLLDPE precursor films with different orientations during uniaxial tensile deformation are schematically illustrated in Figure 7. Specifically, for less oriented precursor films, the lamellae are randomly oriented or slightly oriented along the machine direction. In the linear elastic region I ($0<\varepsilon <\varepsilon_{Y1}$), the increase of $L_{m}$ and $L_{d}$ as well as the rapid decrease of $O_{LJ}$ suggests the lamellae separation and their rearrangement towards the tensile direction (Figure 4b). The slight decrease in the total crystallinity χ is ascribed to the breaking or melting of imperfect lamellas. In region II ($\varepsilon_{Y1}<\varepsilon <\varepsilon_{H}$), the polymorphic transition occurs after the first yield point. While the amorphous chains tend to orient along the tensile direction, this, in turn, stimulates the slippage of lamellae with certain angles relative to the tensile direction. The gradual decrease of χ, together with the increase of $L_{m}$ and $L_{d}$, indicates that the initiation of lamellae tilting or shearing and the rotation of lamellae stack along the tensile direction occur in this region [45,46]. In region III, the normal direction of the lamellae stacks aligns with the diagonal direction. The rapid increase of $L_{m}$ around $\varepsilon_{H}$ suggests that the lamellae are separating from each other continuously. The existence of a maximum in $L_{001}$ and a minimum in $\varepsilon_{001}$ around $\varepsilon_{H}$ implies the maximum shearing or tilting of the crystals [17,47,48]. We get the meridian long period $L_{m}$ until $\varepsilon_{H}$ due to the rotation of the lamellae stacks. The long period along the diagonal direction $L_{d}$ shows the whole information about the long period during the lamellae shifting from meridian to diagonal and then off-equatorial [49]. The large decreases in χ and $L_{200}$, as well as the broader 2θ peak width of the ${\left(200\right)}_{O}$ crystallographic plane, demonstrate the breakdown of the crystals due to the pulling out of the chains or partial melting of small and unstable crystals. A small amount of fibrillar structure might come into being in this region. The second yield point in the stress–strain curve is a result of two concomitant mechanisms. The first is the melting or destruction of the initial lamellae, evidenced by the gaps in $L_{m}$, $L_{d}$ around $\varepsilon_{Y2}$ as well as the reduction in χ and the broadening of the 2θ peaks of all crystallographic planes in the orthorhombic and monoclinic phases. The second is the recrystallization process of the lamellae or crystals in the form of small blocks. In region IV ($\varepsilon >\varepsilon_{Y2}$), recrystallization continues, and the strain hardening happens at large strains. The plateaus in $L_{m}$, $L_{d}$, χ, $\chi_{O}$, and $\chi_{M}$ suggest the formation of the fibrillar structure.

For the highly-oriented precursor film PE-87, the step-cyclic deformation experiments display its hard elastic property due to the highly-oriented lamellae stacks. Similar to the less oriented precursor films, four deformation regions can be observed. Due to the high orientation, the structure transitions or the deformation mechanism overlap or get delayed. In region I, the increases in the long periods $L_{m}$ and $L_{d}$ and the decrease in $O_{LJ}$ signify the initiation of lamellae separation as the stretching begins (Figure 5e). As χ remains almost constant in this region, it implies a few unstable crystals or lamellae fracture and tries to reorient towards the tensile direction. The slight decrease in $O_{LJ}$ suggests that the lamellae stacks do not intend to lose their initial orientations. The initial decrease in $L_{200}$ is possible due to the high orientation of the ${\left(200\right)}_{O}$ crystallographic plane. The continuous increases in the long periods $L_{m}$ and $L_{d}$ together with the monotonic decrease in $O_{LJ}$ indicate a larger separation between lamellae. The formation of the monoclinic crystal originates from the slipping or shearing in a few crystal planes, during which χ decreases gradually. In particular, the monoclinic fraction in PE-87 is very low as compared with less oriented precursor films due to the tightly-packed lamellae. The increases in $L_{m}$ and $L_{d}$ suggest the stress-induced micro-phase separation. The formation of fibrillar structure might stimulate the lamellae rotation or tilting and reorientation in this region. Therefore, the stress distributes more homogeneously on the surface of crystalline lamellae, and the cohesive strength of crystals is drastically reduced compared to the amorphous phase as evidenced by the decreasing χ, $L_{200}$ and $L_{001}$. Here, the normal direction of lamellae stacks is roughly along the diagonal direction, while the amorphous chains tend to arrange along the tensile direction. With an increase in the strain around the second yield point, the lamellae break down into crystal fibers as a result of melting-recrystallization. The step-cyclic test verifies the critical point C around the second yield point (see Figure S5b), where the stress on the crystalline lamellae reaches a critical value [50]. Afterward, the lamellae become unstable and the melting-recrystallization process occurs. Further stretching at the final strain hardening stage leads to the slipping of the crystal fibers as they are preferentially oriented along the tensile direction.

## 5. Conclusions

The microstructural evolutions of mLLDPE precursor films with different orientations have been investigated by in situ X-ray scattering techniques. The mechanical properties, especially the double yield behaviors of mLLDPE precursor films were coupled with the orientation, slippage, and fragmentation of crystalline lamellae, as well as the polymorphic transition and melting-recrystallization behavior. The dominating microstructural evolution differs for mLLDPE precursor films with different orientations. For less oriented mLLDPE precursor films, i.e., PE-61, PE-72, and PE-78, the rearrangement of lamellae and the polymorphic transition are responsible for the appearance of the first yield point. As the strain increases further, the fragmentation of lamellae and the accompanying melting-recrystallization occur. In comparison, the rearrangement of crystalline lamellae is not observed for highly-oriented mLLDPE precursor film PE-87, and only the polymorphic transition leads to the first yield point; After that, the formation of the fibrillar structure caused by the slippage of lamellae accounts for the occurrence of the second yield point.

The present work has demonstrated that the double yield behavior observed in mLLDPE precursor films under uniaxial tensile deformation mainly stems from the rearrangement and fragmentation of lamellae at sub-micron length scale, and the polymorphic transition at the nanoscale. However, the mechanical behavior of semi-crystalline polymers is not solely determined by the microstructure at the length scales detected by SAXS/WAXS. Structural information during the tensile formation at a micro or larger length scales might be closely related to the double yield phenomenon, which can be acquired by the ultra-small-angle X-ray scattering (USAXS) in the future.

## Figures and Tables

**Table 1:** Structural information of mLLDPE precursor films.

Draw Ratio	$O_{\mathrm{LJ}}$	χ (%)	$L_{m}$ (nm)	$L_{d}$ (nm)	Sample Name
20	0.61	41.5	20.56	20.76	PE-61
80	0.72	49.9	17.94	19.82	PE-72
120	0.78	49.1	16.63	19.59	PE-78
240	0.87	44.9	17.32	18.00	PE-87

## Data Availability

Data is contained within the article or supplementary material.

## Supplementary Materials

The following are available online at https://www.mdpi.com/2073-4360/13/1/126/s1, Figure S1: 2D WAXS patterns for samples with different orientations during uniaxial tensile deformation, Figure S2: Representative 1D SAXS intensity profiles along the (a) meridian direction, (b) diagonal direction, and (c) equatorial direction, Figure S3: Azimuthal integrations for PE-72, PE-78, and PE-87 with the increasing strain, Figure S4: (a) Example of the multiple-peak fit of the 1D integrated WAXS intensity curve with Gaussian functions to represent the amorphous, orthorhombic, and monoclinic crystal phases. (b) Representative 2D WAXS pattern marks the detailed orthorhombic crystal and monoclinic crystal lattice plane diffractions, Figure S5: (a) step-cyclic deformation stress–strain curves for PE-87; (b) reversible part ε(H.c) and irreversible part ε(H.b) of the total strain ε(H) as a function of the stress. The locations of the critical points A, B, and C are indicated, Figure S6: The experimental device setup for the WAXS measurement carried out at the beamline BL19U2 in the Shanghai Synchrotron Radiation Facility (SSRF).

## Abbreviations

The following abbreviations are used in this manuscript:

mLLDPE	Metallocene linear low-density polyethylene
SAXS	Small-angle X-ray scattering
WAXS	Wide-angle X-ray scattering
