# Supplementary material for: Microstructural Origin of the Double Yield Points of the Metallocene Linear Low-Density Polyethylene (mLLDPE) Precursor Film under Uniaxial Tensile Deformation
Source: Polymers (Basel). 2020 Dec 30;13(1):126. doi: 10.3390/polym13010126 (PMC7794766; doi:10.3390/polym13010126)
Supplement: Supplementary file 1 [file polymers-13-00126-s001.pdf]

polymers

MDPI

Supplementary Materials: Microstructural Origin of the Double Yield Points of the Metallocene Linear Low-Density Polyethylene (mLLDPE) Precursor Film under Uniaxial Tensile Deformation

Obaid Iqbal, Jean Claude Habumugisha, Shengyao Feng, Yuanfei Lin, Wei Chen, Wancheng Yu and Liangbin Li

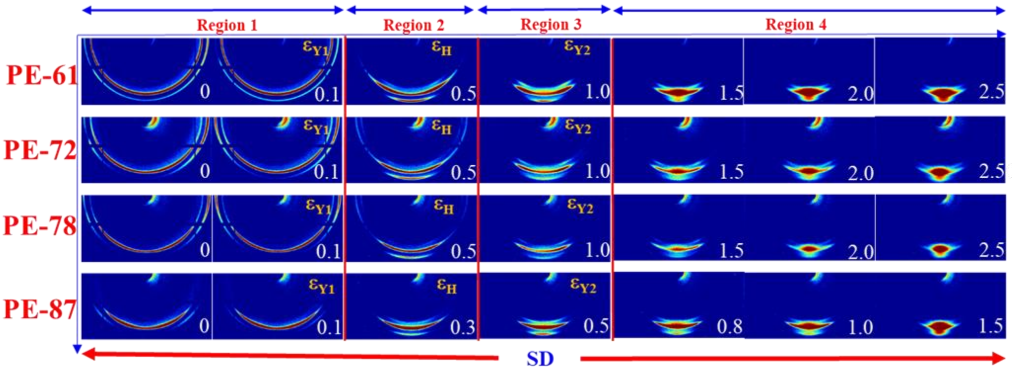

Figure S1. 2D WAXS patterns for samples with different orientations during uniaxial tensile deformation

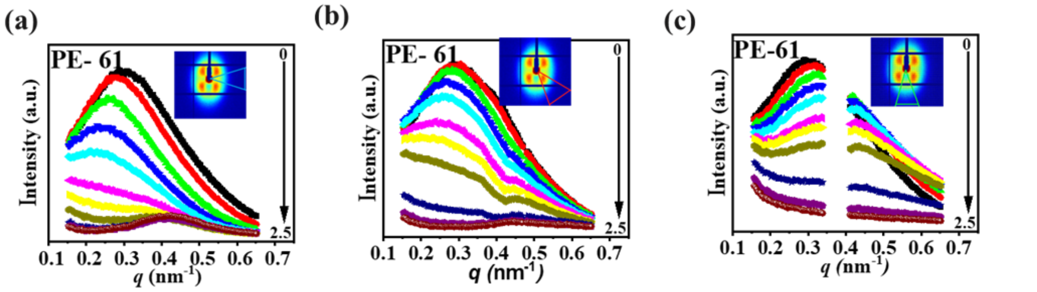

Figure S2. Representative 1D SAXS intensity profiles along the (**a**) meridian direction, (**b**) diagonal direction, and (**c**) equatorial direction Section S3: Morphological characteristics of filter webs.

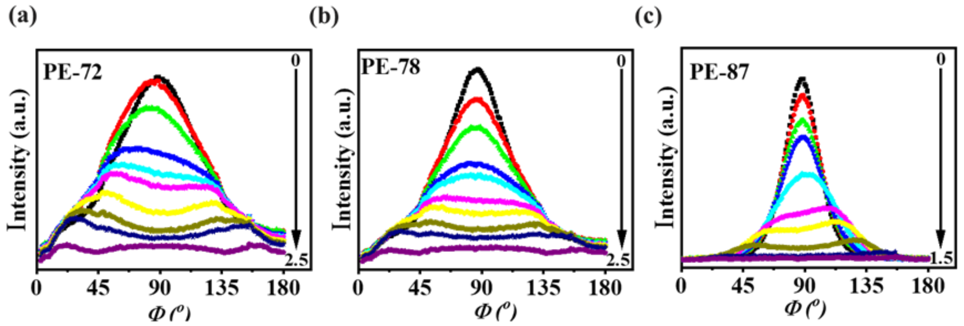

Figure S3. Azimuthal integrations for PE-72, PE-78, and PE-87 with the increasing strain

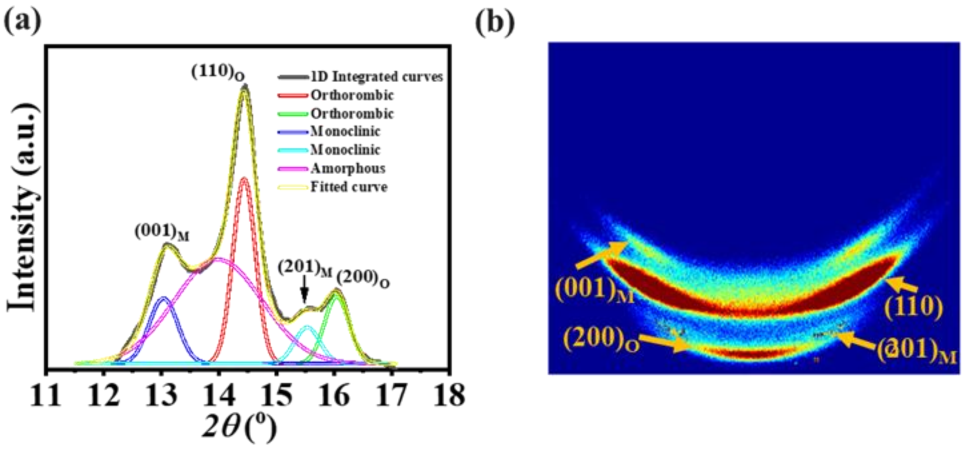

Figure S4. (**a**) Example of the multiple-peak fit of the 1D integrated WAXS intensity curve with Gaussian functions to represent the amorphous, orthorhombic, and monoclinic crystal phases. (**b**) Representative 2D WAXS pattern marks the detailed orthorhombic crystal and monoclinic crystal lattice plane diffractions.

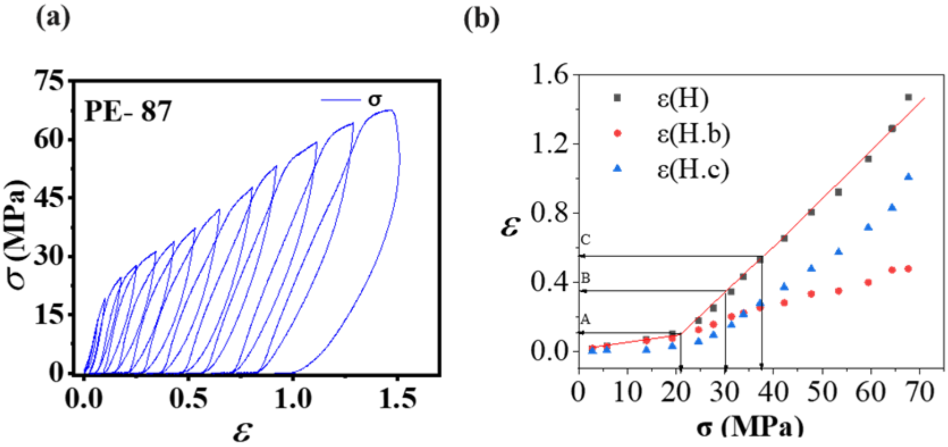

Figure S5. (**a**) Step-cyclic deformation stress–strain curves for PE-87; (**b**) reversible part ε (*H.c*) and irreversible part ε (*H.b*) of the total strain ε (*H*) as a function of the stress. The locations of the critical points A, B, and C are indicated

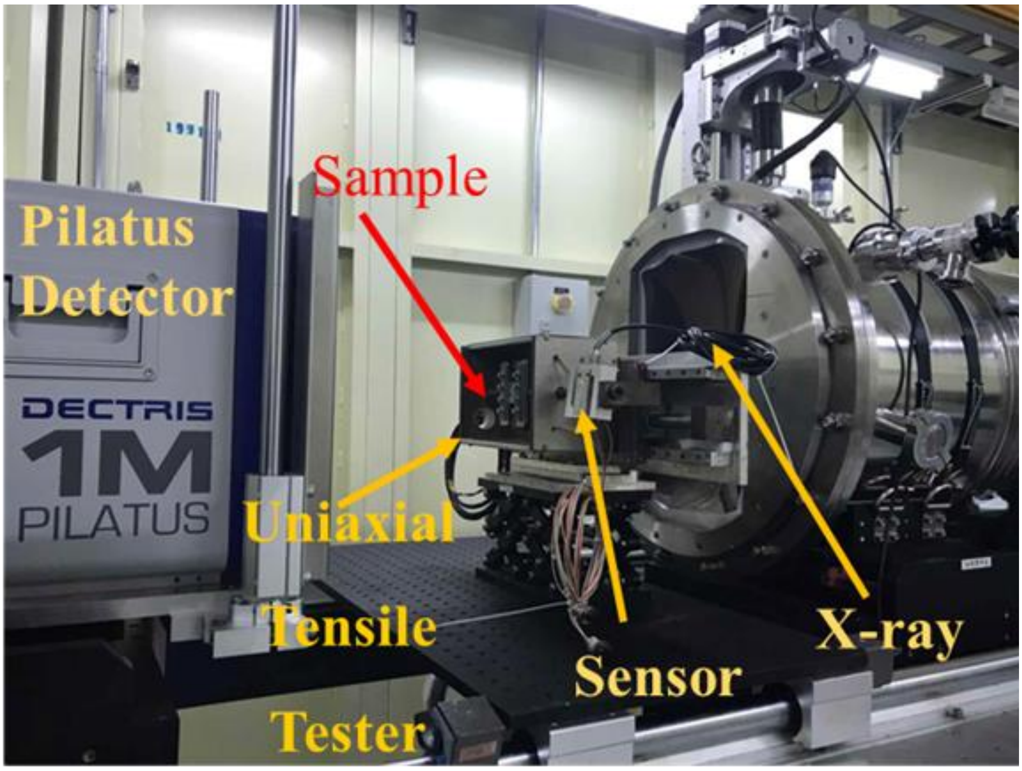

Figure S6. The experimental device setup for the WAXS measurement carried out at the beamline BL19U2 in the Shanghai Synchrotron Radiation Facility (SSRF)
